# How an Enterolith May Behave

**Published:** 1902-12

**Authors:** T. M. McIntosh

**Affiliations:** Thomasville, Ga.


### HOW AN ENTEROLITH MAY BEHAVE.

By T. M. McINTOSH, M.D.,
Thomasville, Ga.

Case 1.—L. J. P., Florida; white, multipara; aged forty-eight years. Residing in south Georgia and Florida all her life, but last five years in north Georgia. Was telegraphed to come to operate for obstruction of the bowels. Arriving about two hours before daylight, and the patient being quiet from a hypodermic of morphine, we went to bed to wait for day to operate. After some time we were awakened to tell us that patient had had a free movement from the bowels, and passed a large "lump," which on examination we found to be an enterolith one and seven-eighth inches long and one and one-eighth inches in diameter.

The history was that for two years she had been subject to sudden and severe attacks of abdominal pain, much nausea and vomiting, with much difficulty of moving the bowels, but when they did move there was relief. None of the attacks had been attended with fever.

The present attack, coming on suddenly, with abdominal pain, nausea, vomiting and obstinate constipation, two days before the attending physician had treated with morphia hypodermically for pain and some purgatives, and large warm-water enemas. She was relieved by bowel action.

Case 2.—Mr. L., aged thirty-eight years, white, Tallahassee, Florida. Had moved from a northern State two years before. I was telegraphed to come to operate for appendicitis. On arrival found patient with fast and feeble pulse, high fever, hiccough, enormously distended abdomen. This condition had come on as a result of what patient thought was "colic" five days before. During a period of three months there had been several other attacks of "colic," which had been relieved without medical aid, and as it was thought the present attack would get well as the others had done, there was delay in sending for a physician and a rejection of his advice to operate until the present grave condition resulted. It was not an inviting case for operation, but, with T. Gaillard Thomas, we think it "wicked" to operate for brilliant statistics, we decided to open the abdomen and give the patient at least a chance. Opening the abdomen, there was found quite a collection of pus, two enteroliths the size of half a peanut, which had ulcerated through the appendix. The appendix, soft and friable, was tied off, the wound kept open by drain and gauze. Death in forty-eight hours.

CASE 3.—Miss F., aged twenty years, white. Resident of Florida all her life. In April, 1900, was seized with sudden, severe pain in right iliac region. The acute pain ceased after several hours over the right iliac area, then was easily made out a distinct and circumscribed swelling, that was tender and painful on pressure. Coincident with beginning of the attack there was fever, which continued for a month. Nine months later there was another attack, except that there was fever but a few days. From this attack till January, 1902, there had been several mild attacks. From this last date there was more or less continued pain in the iliac region, and the swelling was distinctly felt, tender on pressure. There was at no time complete freedom from pain or uneasy sensation. The bowels were habitually constipated. The physician who had treated her in these attacks, and a consultant, had diagnosed appendicitis. In April, 1902, there was quite a severe acute attack, pain, nausea, fever. May 13th she was sent to me. Over McBurney's point a distinct tumor, painful on pressure, could be made out and a diagnosis of recurring appendicitis made. Preparatory to operation the bowels were moved daily, and only animal broths given as food for three days prior to operation, which was done May 18th.

The muscle-splitting incision was made. Passing the finger into abdominal cavity, the appendix was readily found and, with the cecum, withdrawn through the incision. The appendix was perfectly normal, free from the least evidence of present or past inflammation, but in the cecum, up against the valve, there was an impacted mass which, on dislodgment, separated into three distinct bodies, the largest the size of a hickory-nut, the others twice and three times smaller.

These masses were pushed through the ileocecal valve with the finger, by invaginating the bowels, as it were. The appendix was let alone. In forty-eight hours salts was given, and with the first bowel movement the largest mass came, and with the second the other two.

They were enteroliths of irregular shapes, somewhat faceted, where they seemed to have been in contact while within the bowel. Recovery was prompt and uneventful. It was suggested to me during the operation to remove the appendix, though healthy, as a precaution against a possible future appendicitis, but it was thought a good principle in surgery, as well as medicine, to do only what diseased conditions called for.

Kelly, of Baltimore, in an October, 1902, issue of the *Journal of the American Medical Association,* has collected the opinions of some of the leading surgeons on this point. He says: "The opinion of the majority of the surgeons in this country is against the removal of a perfectly healthy appendix, 44 to 26 being the proportion shown in my investigations."

The questions he propounded were: "When the abdomen is opened for other causes, and the perfectly normal appendix easily accessible, is it your rule to remove it? No, 44. Yes, 26." "When the appendix is slightly adherent to neighboring structures, peritoneum, ovarian or fibroid tumors, do you then remove it? Yes, 60. No, 7." Kelly himself does not remove the normal appendix.

An enterolith is not by any means an infrequent formation in the bowels, nor an infrequent cause of an obstruction or an appendicitis, but these cases under my observation, producing quite different conditions, were of interest, and especially the last case, where all the symptoms of a recurring and long-existing appendicitis were present, and accompanied by fever, which, in such cases, we deem to be the expression of an inflammation, but which proved not to be an appendicitis, and the length of time the foreign body remained in the cecum and producing no symptoms of obstruction.

